# Entropy, Graph Homomorphisms, and Dissociation Sets

**DOI:** 10.3390/e25010163

**Published:** 2023-01-13

**Authors:** Ziyuan Wang, Jianhua Tu, Rongling Lang

**Affiliations:** 1 School of Mathematics and Statistics, Beijing Technology and Business University, Beijing 100048, China; 2 School of Electronics and Information Engineering, Beihang University, Beijing 100191, China

**Keywords:** entropy, graph homomorphisms, dissociation sets, independent sets, bipartite graphs

## Abstract

Given two graphs *G* and *H*, the mapping of f:V(G)→V(H) is called a graph homomorphism from *G* to *H* if it maps the adjacent vertices of *G* to the adjacent vertices of *H*. For the graph *G*, a subset of vertices is called a dissociation set of *G* if it induces a subgraph of *G* containing no paths of order three, i.e., a subgraph of a maximum degree, which is at most one. Graph homomorphisms and dissociation sets are two generalizations of the concept of independent sets. In this paper, by utilizing an entropy approach, we provide upper bounds on the number of graph homomorphisms from the bipartite graph *G* to the graph *H* and the number of dissociation sets in a bipartite graph *G*.

## 1. Introduction

Throughout this paper, we consider only undirected and labeled graphs which contain no multiple edges. Let *G* be a simple graph. For the vertex v∈V(G), let N(v)={u|uv∈E(G)} and the degree d(v) of *v* be the size of N(v). The graph *G* is regular if all vertices have the same degree; if this degree is *d*, then G is *d*-regular. A subset of the vertices of *G* is called an independent set if it induces a subgraph of *G* containing no edges. The empty set is also thought to be an independent set of *G*. Let

I(G)={I|IisanindependentsetofG},

and

i(G)=|I(G)|.

If the vertex set V(G) of *G* can be partitioned into two nonempty independent sets *L* and *R*, so that L∪R=V(G) and L∩R=∅, then *G* is a bipartite graph and is denoted by G[L,R]. Furthermore, if all vertices in *L* or *R* have the same degree, then *G* is called a *half-regular* bipartite graph. For a positive integer *k*, the disjoint union of the *k* copies of *G* is denoted by k·G.

In the last decades, the problem of upper bounding the number of discrete structures satisfying specific properties has received considerable attention. In particular, there have been a lot of results on upper bounding the number of independent sets in a given class of graphs. Using an entropy approach, Kahn [1] obtained the greatest number of independent sets in regular bipartite graphs. Zhao [2] extended Kahn’s result to all regular graphs.

**Theorem 1.**  *[1,2] If G is an n-vertex d-regular graph, then*

$$i\left(G\right)\leq {\left(2^{d+1}-1\right)}^{\frac{n}{2d}},$$

*with equality if and only if n is divisible by 2d and $G≅\frac{n}{2d}\cdot K_{d,d}$.*

The result in Theorem 1 can be rephrased as: if *G* is a *d*-regular graph, then

(1) $$i\left(G\right)\leq \prod_{uv\in E(G)}{\left(2^{d(u)}+2^{d(v)}-1\right)}^{1/(d(u)d(v))}=\prod_{uv\in E(G)}{\left(i\left(K_{d(u),\;d(v)}\right)\right)}^{1/(d(u)d(v))}.$$

Kahn [1] conjectured that the inequality (1) also holds for any graph *G* that contains no isolated vertices. In 2019, Sah et al. [3] solved the conjecture.

**Theorem 2.**  *[3] If G is a graph that contains no isolated vertices, then*

$$i\left(G\right)\leq \prod_{uv\in E(G)}{\left(2^{d(u)}+2^{d(v)}-1\right)}^{1/(d(u)d(v))}=\prod_{uv\in E(G)}{\left(i\left(K_{d(u),\;d(v)}\right)\right)}^{1/(d(u)d(v))}.$$

Recently, Sason [4] presented an entropy approach proof of Theorem 2 under the assumption that the graph is a half-bipartite graph.

For the extremal problem of this kind, other special graph substructures, such as maximal (maximum) independent sets [5,6,7], matchings [8], minimal dominating sets [9], maximum dissociation sets [10], etc., were also studied by the researchers.

In this paper, we focus on two generalizations of the concept of independent sets. The first is graph homomorphism. Given two graphs *G* and *H*, the mapping f:V(G)→V(H) is called a graph homomorphism from *G* to *H* if it maps the adjacent vertices of *G* to the adjacent vertices of *H*. Let

Hom(G,H)={f:V(G)→V(H):f(u)f(v)∈E(H)∀uv∈E(G)},

and

hom(G,H)=|Hom(G,H)|.

The graph *G* is called the source graph and is usually simple; the graph *H* is called the target graph and it is allowed to have loops. For a simple graph *G*, when *H* is a graph with $V\left(H\right)=\left\{v_{1},v_{2}\right\}$ and $E\left(H\right)=\left\{v_{1}v_{1},v_{1}v_{2}\right\}$, for any f∈Hom(G,H), the vertex set

$$\left\{u:u\in V\left(G\right)\;\mathrm{and}\;f\left(u\right)=v_{2}\right\}$$

is an independent set of *G*, and it is easy to see that there exists a bijection between the elements of Hom(G,H) and the independent sets of *G*. Galvin and Tetali [11] extended the result in Theorem 1 to graph homomorphisms as follows.

**Theorem 3.**  *[11] Let G be a simple d-regular bipartite graph. Then, for any graph H,*

$$hom\left(G,H\right)\leq {\left[hom\left(K_{d,\;d},H\right)\right]}^{\left|V(G)|/(2d\right)}=\prod_{uv\in E(G)}{\left[hom\left(K_{d,\;d},H\right)\right]}^{1/d^{2}}.$$

It can be shown that the hypothesis in Theorem 3 that *G* is a bipartite graph cannot be discarded [12]. Galvin [13] posed the following conjecture that extends Theorem 3.

**Conjecture 1.**  *[13] Let G be a simple bipartite graph that contains no isolated vertices. Then, for any graph H,*

$$hom\left(G,H\right)\leq \prod_{uv\in E(G)}{\left[hom\left(K_{d(u),\;d(v)},H\right)\right]}^{1/(d(u)d(v))}.$$

The first contribution of our work is to prove that Conjecture 1 holds for simple half-regular bipartite graphs. Let *G* be a simple bipartite graph that contains no isolated vertices. We obtain an upper bound on hom(G,H) for any graph *H* using an entropy approach.

**Theorem 4.**  *Let G[L,R] be a simple bipartite graph that contains no isolated vertices. For any vertex v∈R, let $\delta_{v}:=\min_{u\in N(v)}\left\{d\left(u\right)\right\}$. Then, for any graph H,*

*$hom\left(G,H\right)\leq \prod_{uv\in E(G),u\in L,v\in R}{\left[hom\left(K_{\delta_{v},\;d\left(v\right)},H\right)\right]}^{\frac{1}{\delta_{v}d\left(v\right)}}$.*

The following corollary can be easily deduced from Theorem 4 and implies that Conjecture 1 holds for simple half-regular bipartite graphs.

**Corollary 1.**  *Let G be a simple half-regular bipartite graph that contains no isolated vertices. Then, for any graph H,*

$$hom\left(G,H\right)\leq \prod_{uv\in E(G)}{\left[hom\left(K_{d(u),\;d(v)},H\right)\right]}^{1/(d(u)d(v))}.$$

The second generalization of the concept of independent sets considered in this paper is dissociation sets. Let *G* be a simple graph. A dissociation set of *G* is a set of vertices which induces a subgraph containing no paths of order 3, i.e., a subgraph of a maximum degree which is at most one. Clearly, an independent set of *G* is also a dissociation set of *G*. Let

D(G)={D|DisadissociatiosetofG},

and

Φ(G)=|D(G)|.

In the early 1980s, Yannakakis [14] introduced the concept of dissociation sets and proved that the problem of finding a dissociation set of the largest possible size in a given graph is NP-complete in bipartite graphs. The problem is also NP-complete in planar graphs of a maximum degree which is at most four [15].

The second contribution of our work is to give an upper bound on Φ(G) for the simple bipartite graph *G* by the entropy approach.

**Theorem 5.**  *Let G[L,R] be a simple bipartite graph that contains no isolated vertices. For any vertex v∈R, let $\delta_{v}:=\min_{u\in N(v)}\left\{d\left(u\right)\right\}$. We have*

$$\Phi \left(G\right)\leq \prod_{uv\in E(G),u\in L,v\in R}{\left(\left(d\left(v\right)+1\right)\cdot 2^{\delta_{v}}+2^{d(v)}-d\left(v\right)-1\right)}^{\frac{1}{\delta_{v}d\left(v\right)}}.$$

The following corollary can be easily obtained from Theorem 5.

**Corollary 2.**  *Let G be an n-vertex simple d-regular bipartite graph. Then,*

$$\Phi \left(G\right)\leq {\left(\left(d+2\right)\cdot 2^{d}-d-1\right)}^{\frac{n}{2d}}.$$

The rest of this paper is organized as follows. In Section 2, we introduce some of the basic concepts and notations of entropy, as well as several important preliminary lemmas. In Section 3, the proofs of Theorems 4 and 5 are presented. The upper bound given in Corollary 2 is not tight. When d=2, a simple two-regular bipartite graph is a disjoint union of the even cycles. In Section 4, we give a tight upper bound on Φ(G) for a simple two-regular graph *G*. In Section 5, we summarizes our work.

## 2. Entropy

All the preliminary lemmas introduced in this section and their proofs can be found in [16]. Hereinafter, let *X*, *Y*, etc., be discrete random variables. We write p(x) and p(x|y) to denote Pr[X=x] and Pr[X=x|Y=y], respectively. The entropy of the random variable *X* is defined by

$$H\left(X\right)=E\left[\log\frac{1}{p(x)}\right]=\sum_{x}p\left(x\right)\log\frac{1}{p(x)},$$

where the logarithm is base two and we assume that $0\log\frac{1}{0}=0$. It is useful for us to understand entropy H(X) as a measure of the degree of randomness of *X*.

**Lemma 1.**  *If X takes its values on a finite set X, then*

H(X)≤log|X|,

*with equality if and only if X is uniform on X.*

The conditional entropy H(Y|X) of *Y* given *X* and the joint entropy H(X,Y) are defined by

$$H\left(Y\;|\;X\right)=\sum_{x}p\left(x\right)\sum_{y}p\left(y\;|\;x\right)\log\frac{1}{p(y\;|\;x)}=\sum_{x}p\left(x\right)H\left(Y\;|\;X=x\right),$$

and

$$H\left(X,Y\right)=\sum_{x,y}p\left(x,y\right)\log\frac{1}{p(x,y)},$$

respectively. If *Y* is a function of *X*, then we say *X* determines *Y*.

**Lemma 2.**  *(Dropping rule) (1) H(Y|X,Z)≤H(Y|X)≤H(Y);*

*(2) If X determines Z, then H(Y|X)≤H(Y|Z).*

**Lemma 3.**  *(Chain rule)*

H(X,Y)=H(X)+H(Y|X).

*as a general rule, for a random vector $X=(X_{1},X_{2},\cdots ,X_{n})$,*

$$H\left(X\right)=H\left(X_{1}\right)+H\left(X_{2}\;|\;X_{1}\right)+\cdots +H\left(X_{n}\;|\;X_{1},\cdots ,X_{n-1}\right).$$

**Lemma 4.**  *(Subadditivity) For a random vector $\left(X_{1},X_{2},\cdots ,X_{n}\right)$,*

$$H\left(X_{1},X_{2},\cdots ,X_{n}\right)\leq \sum_{i=1}^{n}H\left(X_{i}\right),$$

*and*

$$H\left(X_{1},X_{2},\cdots ,X_{n}\;|\;Y\right)\leq \sum_{i=1}^{n}H\left(X_{i}\;|\;Y\right).$$

## 3. Proofs of Theorems 4 and 5

**Proof of Theorem 4.**  We first introduce a useful expression for $hom(K_{m,n},H)$ that was given in [11]. Consider a complete bipartite graph $K_{m,n}$ with bipartition (U,V). For A⊆V(H), let

T(V,A)={f:V→A:fsurjective}

and

C(A)={w∈V(H):wz∈E(H)∀z∈A}.

Then,

(2) $$hom\left(K_{m,n},H\right)=\sum_{A\subseteq V(H)}|T\left(V,A\right)||C\left(A\right){|}^{m}.$$

Let ℓ:=|L| and r:=|R|. We assign the labels $u_{1},u_{2},\cdots ,u_{\ell }$ to the vertices of *L* and the labels $v_{1},v_{2},\cdots ,v_{r}$ to the vertices of *R*.

Choose a graph homomorphism *f* uniformly at random from Hom(G,H). For S⊆V(G), we write $f_{S}$ for the restriction of *f* to *S*. When S={v}, we write $f_{v}$ for $f_{\left\{v\right\}}$. For v∈V(G) and A⊆V(H), let $M_{v}:=\left\{f\left(u\right),u\in N\left(v\right)\right\}$, and $m_{v}\left(A\right):=\mathrm{Pr}\left[M_{v}=A\right]$. Clearly, for every vertex v∈V(G),

(3) $$\sum_{A\subseteq V(H)}m_{v}\left(A\right)=1.$$

By Lemmas 1 and 3, we have

(4) H(f)=loghom(G,H)

and

(5) $$H\left(f\right)=H\left(f_{L}\right)+H\left(f_{R}\;|\;f_{L}\right).$$

We will prove that

(6) $$H\left(f_{L}\right)\;\leq \;\sum_{u\in L}\sum_{v\in N(u)}\frac{1}{\delta_{v}}\frac{1}{d(v)}H\left(f_{N(v)}\right).$$

By Lemma 3, we have

$$\begin{array}{cc} H(f_{L}) & =H\left(f_{u_{1}}\right)+H\left(f_{u_{2}}\;|\;f_{u_{1}}\right)+\cdots +H\left(f_{u_{\ell }}\;|\;f_{u_{1}},\cdots ,f_{u_{\ell -1}}\right)\\ \; & =\sum_{i=1}^{\ell }H\left(f_{u_{i}}\;|\;f_{u_{1}},\cdots ,f_{u_{i-1}}\right). \end{array}$$

Suppose that for a vertex v∈R, $N\left(v\right)=\left\{u_{i_{1}},\cdots ,u_{i_{k}}\right\}$, where $1\leq i_{1}<\cdots <i_{k}\leq \ell .$ Then, by Lemmas 2 and 3,

$$\begin{array}{cc} H(f_{N(v)}) & =H\left(f_{u_{i_{1}}}\right)+H\left(f_{u_{i_{2}}}\;|\;f_{u_{i_{1}}}\right)+\cdots +H\left(f_{u_{i_{k}}}\;|\;f_{u_{i_{1}}},f_{u_{i_{2}}},\cdots ,f_{u_{i_{\left(k-1\right)}}}\right)\\ \; & \geq H\left(f_{u_{i_{1}}}\;|\;f_{u_{1}},\cdots ,f_{u_{i_{1}-1}}\right)+H\left(f_{u_{i_{2}}}\;|\;f_{u_{1}},\cdots ,f_{u_{i_{2}-1}}\right)+\cdots \\ \; & \;+H(f_{u_{i_{k}}}\;|\;f_{u_{1}},\cdots ,f_{u_{i_{k}-1}}). \end{array}$$

Recall that for a vertex v∈R, $\delta_{v}=\min_{u\in N(v)}\left\{d\left(u\right)\right\}$. For any vertex u∈L,

$$\sum_{v\in N(u)}\frac{1}{\delta_{v}}\geq \sum_{v\in N(u)}\frac{1}{d(u)}=1.$$

Thus, we have

$$\begin{array}{cc} \sum_{u\in L}\sum_{v\in N(u)}\frac{1}{\delta_{v}}\frac{1}{d(v)}H\left(f_{N(v)}\right) & =\sum_{v\in R}d\left(v\right)\cdot \frac{1}{\delta_{v}}\frac{1}{d(v)}H\left(f_{N(v)}\right)=\sum_{v\in R}\frac{1}{\delta_{v}}H\left(f_{N(v)}\right)\\ \; & \geq \sum_{i=1}^{\ell }\sum_{v\in N(u_{i})}\frac{1}{\delta_{v}}H\left(f_{u_{i}}\;|\;f_{u_{1}},\cdots ,f_{u_{i-1}}\right)\\ \; & \geq \sum_{i=1}^{\ell }H\left(f_{u_{i}}\;|\;f_{u_{1}},\cdots ,f_{u_{i-1}}\right)\\ \; & =H(f_{L}). \end{array}$$

Now we have proved that the inequality (6) holds. Furthermore, since $H\left(f_{N(v)}\right)\leq H\left(f_{N(v)},M_{v}\right)=H\left(M_{v}\right)+H\left(f_{N(v)}\;|\;M_{v}\right),$

(7) $$H\left(f_{L}\right)\leq \sum_{u\in L}\sum_{v\in N(u)}\frac{1}{\delta_{v}}\frac{1}{d(v)}H\left(f_{N(v)}\right)\;\leq \;\sum_{u\in L}\sum_{v\in N(u)}\frac{1}{\delta_{v}}\frac{1}{d(v)}\left[H\left(M_{v}\right)\;+\;H\left(f_{N(v)}\;|\;M_{v}\right)\right].$$

Next, consider $H(f_{R}\;|\;f_{L})$.

(8) $$\begin{array}{cc} H(f_{R}\;|\;f_{L}) & \leq \sum_{v\in R}H\left(f_{v}\;|\;f_{L}\right) \end{array}$$

(9) $$\begin{array}{cc} \; & =\sum_{u\in L}\sum_{v\in N(u)}\frac{1}{d(v)}H\left(f_{v}\;|\;f_{L}\right) \end{array}$$

(10) $$\begin{array}{cc} \; & \leq \sum_{u\in L}\sum_{v\in N(u)}\frac{1}{d(v)}H\left(f_{v}\;|\;f_{N(v)}\right) \end{array}$$

(11) $$\begin{array}{cc} \; & \leq \sum_{u\in L}\sum_{v\in N(u)}\frac{1}{d(v)}H\left(f_{v}\;|\;M_{v}\right), \end{array}$$

where the inequality (8) follows from Lemma 4, the inequality (10) follows from Lemma 2 and the fact that for any vertex v∈R, N(v)⊆L, and the inequality (11) follows from the fact that $f_{N(v)}$ determines $M_{v}$.

Combining (5)–(11), we have

(12) $$\begin{array}{cc} H(f) & =H\left(f_{L}\right)+H\left(f_{R}\;|\;f_{L}\right)\\ \; & \leq \sum_{u\in L}\sum_{v\in N(u)}\frac{1}{\delta_{v}}\frac{1}{d(v)}\left[H\left(M_{v}\right)\;+\;H\left(f_{N(v)}\;|\;M_{v}\right)\right]+\sum_{u\in L}\sum_{v\in N(u)}\frac{1}{d(v)}H\left(f_{v}\;|\;M_{v}\right)\\ \; & =\sum_{u\in L}\sum_{v\in N(u)}\frac{1}{\delta_{v}d\left(v\right)}\left[H\left(M_{v}\right)+H\left(f_{N(v)}\;|\;M_{v}\right)+\delta_{v}H\left(f_{v}\;|\;M_{v}\right)\right].\\ \; & =\sum_{u\in L}\sum_{v\in N(u)}\frac{1}{\delta_{v}d\left(v\right)}\sum_{A\subseteq V(H)}[m_{v}\left(A\right)\log\frac{1}{m_{v}\left(A\right)}+m_{v}\left(A\right)H\left(f_{N(v)}\;|\;M_{v}=A\right)+\\ \; & \;\;\;\;\;\;+\delta_{v}m_{v}\left(A\right)H\left(f_{v}\;|\;M_{v}=A\right)]. \end{array}$$

By Lemma 1,

(13) $$H\left(f_{N(v)}\;|\;M_{v}=A\right)\leq \log\left|T\left(N\left(v\right),A\right)\right|,$$

and

(14) $$H\left(f_{v}\;|\;M_{v}=A\right)\leq \log\left|C\left(A\right)\right|.$$

Combining (12)–(14), we have

(15) $$\begin{array}{cc} H(f) & \leq \sum_{u\in L}\sum_{v\in N(u)}\frac{1}{\delta_{v}d\left(v\right)}\sum_{A\subseteq V(H)}[m_{v}\left(A\right)\log\frac{1}{m_{v}\left(A\right)}+m_{v}\left(A\right)\log\left|T\left(N\left(v\right),A\right)\right|\\ \; & \;\;\;\;\;\;+\delta_{v}m_{v}\left(A\right)\log|C\left(A\right)|]\\ \; & =\sum_{u\in L}\sum_{v\in N(u)}\frac{1}{\delta_{v}d\left(v\right)}\sum_{A\subseteq V(H)}m_{v}\left(A\right)\log\frac{{\left|T\left(N\left(v\right),A\right)||C\left(A\right)\right|}^{\delta_{v}}}{m_{v}\left(A\right)}\\ \; & \leq \;\sum_{u\in L}\sum_{v\in N(u)}\frac{1}{\delta_{v}d\left(v\right)}\log\sum_{A\subseteq V(H)}\left|T\left(N\left(v\right),A\right)|\right|C_{H}{\left(A\right)|}^{\delta_{v}} \end{array}$$

(16) $$\begin{array}{cc} \; & =\sum_{u\in L}\sum_{v\in N(u)}\frac{1}{\delta_{v}d\left(v\right)}\log hom\left(K_{\delta_{v},\;d\left(v\right)},H\right), \end{array}$$

where the inequality (15) follows from the concavity of the function f(x)=logx and the equality (3), the equality (16) follows from the equality (2).

It follows from (4) and (16) that

$$\begin{array}{cc} hom(G,H) & \leq \prod_{u\in L}\prod_{v\in N(u)}hom{\left(K_{\delta_{v},d\left(v\right)},H\right)}^{\frac{1}{\delta_{v}d\left(v\right)}}\\ \; & =\;\prod_{(u,v)\in E(G),u\in L,v\in R}hom{\left(K_{\delta_{v},\;d\left(v\right)},H\right)}^{\frac{1}{\delta_{v}d\left(v\right)}}. \end{array}$$

We complete the proof of Theorem 4. □

**Proof of Theorem 5.**  Choose a dissociation set *S* uniformly at random from D(G). For every vertex u∈L, we define the random variable $X_{u}$ by:

$$X_{u}=\left\{\begin{array}{c} 1,\;\;\mathrm{if}\;u\in S,\\ 0,\;\;\mathrm{if}\;u\notin S. \end{array}\right.$$

For every vertex v∈R, we define the random variable $Y_{v}$ by:

$$Y_{v}=\left\{\begin{array}{c} 1,\;\;\mathrm{if}\;v\in S,\\ 0,\;\;\mathrm{if}\;v\notin S. \end{array}\right.$$

Let $X:=(X_{u_{1}},\ldots ,X_{u_{\ell }})$ and $Y:=(Y_{v_{1}},\ldots ,Y_{v_{r}})$. By Lemmas 1 and 3, we have

(17) H(X,Y)=logΦ(G),

and

(18) H(X,Y)=H(X)+H(Y|X).

Let *v* be a vertex of *R*. We denote by $X_{N(v)}$ a random vector ${\left(X_{u}\right)}_{u\in N(v)}$. Let $Q_{v}:=1\left\{\sum_{u\in N(v)}X_{u}\leq 1\right\}$ and $q_{v}:=\mathrm{Pr}\left[Q_{v}=1\right]$, where 1{E} is the indicator of an random event *E*.

Similarly, we can prove that

(19) $$H\left(X\right)\leq \sum_{u\in L}\sum_{v\in N(u)}\frac{1}{\delta_{v}}\frac{1}{d(v)}H\left(X_{N(v)}\right).$$

Furthermore, since $H\left(X_{N(v)}\right)\leq H\left(X_{N(v)},Q_{v}\right)=H\left(Q_{v}\right)+H\left(X_{N(v)}\;|\;Q_{v}\right),$

(20) $$H\left(X\right)\leq \sum_{u\in L}\sum_{v\in N(u)}\frac{1}{\delta_{v}}\frac{1}{d(v)}H\left(X_{N(v)}\right)\;\leq \;\sum_{u\in L}\sum_{v\in N(u)}\frac{1}{\delta_{v}}\frac{1}{d(v)}\left[H\left(Q_{v}\right)\;+\;H\left(X_{N(v)}\;|\;Q_{v}\right)\right].$$

Next, consider H(Y|X).

(21) $$\begin{array}{cc} H(Y\;|\;X) & \leq \sum_{v\in R}H\left(Y_{v}\;|\;X\right) \end{array}$$

(22) $$\begin{array}{cc} \; & =\sum_{u\in L}\sum_{v\in N(u)}\frac{1}{d(v)}H\left(Y_{v}\;|\;X\right) \end{array}$$

(23) $$\begin{array}{cc} \; & \leq \sum_{u\in L}\sum_{v\in N(u)}\frac{1}{d(v)}H\left(Y_{v}\;|\;X_{N(v)}\right) \end{array}$$

(24) $$\begin{array}{cc} \; & \leq \sum_{u\in L}\sum_{v\in N(u)}\frac{1}{d(v)}H\left(Y_{v}\;|\;Q_{v}\right), \end{array}$$

where the inequality (21) follows from Lemma 4, the inequality (23) follows from Lemma 2 and the fact that for any vertex v∈R, N(v)⊆L, and the inequality (24) follows from the fact that $X_{N(v)}$ determines $Q_{v}$.

Combining (18)–(24), we have

(25) $$\begin{array}{cc} H(X,Y) & =\;H(X)+H(Y\;|\;X)\\ \; & \leq \;\sum_{u\in L}\sum_{v\in N(u)}\frac{1}{\delta_{v}}\frac{1}{d(v)}\left[H\left(Q_{v}\right)+H\left(X_{N(v)}\;|\;Q_{v}\right)\right]+\sum_{u\in L}\sum_{v\in N(u)}\frac{1}{d(v)}H\left(Y_{v}\;|\;Q_{v}\right)\\ \; & =\;\sum_{u\in L}\sum_{v\in N(u)}\frac{1}{\delta_{v}d\left(v\right)}\left[H\left(Q_{v}\right)+H\left(X_{N(v)}\;|\;Q_{v}\right)+\delta_{v}H\left(Y_{v}\;|\;Q_{v}\right)\right]. \end{array}$$

For the random variable $Q_{v}$,

(26) $$H\left(Q_{v}\right)=q_{v}\log\frac{1}{q_{v}}+\left(1-q_{v}\right)\log\frac{1}{1-q_{v}}.$$

Consider the conditional entropy $H(X_{N(v)}\;|\;Q_{v})$. If $Q_{v}=1$, then $X_{u}=1$ for at most one vertex *u* in N(v), so by Lemma 1,

$$H\left(X_{N(v)}\;|\;Q_{v}=1\right)\leq \log\left(d\left(v\right)+1\right).$$

If $Q_{v}=0$, then $X_{u}=1$ for at least two vertices *u* in N(v), so by Lemma 1,

$$H\left(X_{N(v)}\;|\;Q_{v}=0\right)\leq \log\left(2^{d(v)}-d\left(v\right)-1\right).$$

Then,

(27) $$\begin{array}{cc} H(X_{N(v)}\;|\;Q_{v}) & =q_{v}H\left(X_{N(v)}\;|\;Q_{v}=1\right)+\left(1-q_{v}\right)H\left(X_{N(v)}\;|\;Q_{v}=0\right)\\ \; & \leq q_{v}\log\left(d\left(v\right)+1\right)+\left(1-q_{v}\right)\log\left(2^{d(v)}-d\left(v\right)-1\right). \end{array}$$

Consider the conditional entropy $H(Y_{v}\;|\;Q_{v})$. If $Q_{v}=1$, then $Y_{v}$ may be 0 or 1. If $Q_{v}=0$, then $Y_{v}$ must be 0. Thus,

(28) $$\begin{array}{cc} H(Y_{v}\;|\;Q_{v}) & =q_{v}H\left(Y_{v}\;|\;Q_{v}=1\right)+\left(1-q_{v}\right)H\left(Y_{v}\;|\;Q_{v}=0\right)\\ \; & \leq q_{v}\log2. \end{array}$$

It follows from (25)–(28) that

(29) $$\begin{array}{cc} H(X,Y) & \leq \;\sum_{u\in L}\sum_{v\in N(u)}\frac{1}{\delta_{v}d\left(v\right)}[q_{v}\log\frac{1}{q_{v}}+\left(1-q_{v}\right)\log\frac{1}{1-q_{v}}\\ \; & \;\;\;+q_{v}\log\left(d\left(v\right)+1\right)+\left(1-q_{v}\right)\log\left(2^{d(v)}-d\left(v\right)-1\right)+\delta_{v}q_{v}\log2]\\ \; & =\sum_{u\in L}\sum_{v\in N(u)}\frac{1}{\delta_{v}d\left(v\right)}\left[q_{v}\log\frac{\left(d\left(v\right)+1\right)2^{\delta_{v}}}{q_{v}}+\left(1-q_{v}\right)\log\frac{2^{d(v)}-d\left(v\right)-1}{1-q_{v}}\right]\\ \; & \leq \sum_{u\in L}\sum_{v\in N(u)}\frac{1}{\delta_{v}d\left(v\right)}\log\left[\left(d\left(v\right)+1\right)2^{\delta_{v}}+2^{d(v)}-d\left(v\right)-1\right], \end{array}$$

where the inequality (29) follows from the concavity of the function f(x)=logx.

It follows from (17) and (29) that

$$\begin{array}{cc} \Phi (G) & \leq \prod_{u\in L}\prod_{v\in N(u)}{\left[\left(d\left(v\right)+1\right)2^{\delta_{v}}+2^{d(v)}-d\left(v\right)-1\right]}^{\frac{1}{\delta_{v}d\left(v\right)}}\\ \; & =\prod_{(u,v)\in E(G),u\in L,v\in R}{\left[\left(d\left(v\right)+1\right)2^{\delta_{v}}+2^{d(v)}-d\left(v\right)-1\right]}^{\frac{1}{\delta_{v}d\left(v\right)}}. \end{array}$$

We complete the proof of Theorem 5. □

## 4. Further Remarks

By Corollary 2, if *G* is an *n*-vertex simple two-regular bipartite graph, then

$$\Phi \left(G\right)\leq 13^{\frac{n}{4}}\approx 1.8988^{n}.$$

In this section, we give a tight upper bound on Φ(G) for a simple two-regular graph *G*.

**Theorem 6.**  *If G is an n-vertex simple two-regular bipartite graph, then*

$$\Phi \left(G\right)\leq 19^{\frac{n}{6}}\approx 1.8415^{n},$$

*with equality if and only if n is divisible by six and $G≅\frac{n}{6}\cdot C_{6}$.*

**Proof.**  A simple two-regular bipartite graph is a disjoint union of even cycles. It suffices to prove that if n≥4 and n≠6, then

$$\Phi \left(C_{n}\right)<\Phi {\left(C_{6}\right)}^{\frac{n}{6}}=19^{\frac{n}{6}}\approx 1.8415^{n}.$$

**Claim 1.**  *When n≥3, $\Phi \left(P_{n}\right)\leq 1.14\times 1.84^{n}$, where $P_{n}$ is a path on n vertices.*

**Proof of Claim 1.**  $\Phi (P_{3})=7$, $\Phi (P_{4})=13$, $\Phi (P_{5})=24$. It is easy to verify that when 3≤n≤5, $\Phi \left(P_{n}\right)\leq 1.14\times 1.84^{n}$. When n≥6,

$$\begin{array}{cc} \Phi (P_{n})= & \Phi \left(P_{n-1}\right)+\Phi \left(P_{n-2}\right)+\Phi \left(P_{n-3}\right)\\ \leq & 1.14\times 1.84^{n-3}\left(1.84^{2}+1.84+1\right)\\ \leq & 1.14\times 1.84^{n}. \end{array}$$

□

**Claim 2.**  *When n≥7, $\Phi \left(C_{n}\right)\leq 1.0015\times 1.84^{n}$.*

**Proof of Claim 2.** 
$$\begin{array}{cc} \Phi (C_{n})= & \Phi \left(P_{n-1}\right)+\Phi \left(P_{n-3}\right)+2\Phi \left(P_{n-4}\right)\\ \leq & 1.14\times 1.84^{n-1}+1.14\times 1.84^{n-3}+2\times 1.14\times 1.84^{n-4}\\ = & \frac{1.14\times (1.84^{3}+1.84+2)}{1.84^{4}}\times 1.84^{n}\\ \leq & 1.0015\times 1.84^{n}. \end{array}$$

□

By a direct calculation, $\Phi {\left(C_{4}\right)}^{\frac{1}{4}}=11^{\frac{1}{4}}\approx 1.8212$ and $\Phi {\left(C_{6}\right)}^{\frac{1}{6}}=19^{\frac{1}{6}}\approx 1.8415$. When n≥8, $\Phi {\left(G\right)}^{\frac{1}{n}}\leq {\left(1.0015\times 1.84^{n}\right)}^{\frac{1}{n}}\leq 1.0015^{\frac{1}{8}}\times 1.84\approx 1.8404$. It follows that if n≥4 and n≠6, then $\Phi \left(C_{n}\right)<19^{\frac{n}{6}}\approx 1.8415^{n}.$

We complete the proof of Theorem 6. □

**Theorem 7.**  *If G is an n-vertex simple two-regular graph, then*

$$\Phi \left(G\right)\leq 7^{\frac{n}{3}}\approx 1.9130^{n},$$

*with equality if and only if n is divisible by three and $G≅\frac{n}{3}\cdot C_{3}$.*

**Proof.**  It suffices to prove that if n≥4, then

$$\Phi {\left(C_{n}\right)}^{\frac{1}{n}}<\Phi {\left(C_{3}\right)}^{\frac{1}{3}}=7^{\frac{1}{3}}\approx 1.9130.$$

$\Phi {\left(C_{4}\right)}^{\frac{1}{4}}=11^{\frac{1}{4}}\approx 1.8212$, $\Phi {\left(C_{5}\right)}^{\frac{1}{5}}=21^{\frac{1}{5}}\approx 1.8384$, $\Phi {\left(C_{6}\right)}^{\frac{1}{6}}=19^{\frac{1}{6}}\approx 1.8415$. By the proof of Theorem 6, when n≥7, $\Phi {\left(G\right)}^{\frac{1}{n}}\leq {\left(1.0015\times 1.84^{n}\right)}^{\frac{1}{n}}\leq 1.0015^{\frac{1}{7}}\times 1.84\approx 1.8404$.

We complete the proof of Theorem 7. □

## 5. Conclusions

The study of independent sets has had a central place in graph theory. What is the greatest number of independent sets in an *n*-vertex *d*-regular graph? The problem was initially posed by a mathematician, Andrew Granville, who found applications in combinatorial number theory and combinatorial group theory [17]. Since then, the study of counting independent sets in graphs has been a hot topic in graph theory. Some other applications of the study of this kind was provided in [18].

Graph homomorphisms generalize some of the basic concepts of graph theory, for example, independent sets, graph colorings, etc. One may wonder whether many results on counting independent sets can generalize to graph homomorphisms. A well-known conjecture (Conjecture 1) was posed. In this paper, we partially solve the conjecture and show that the conjecture holds for half-regular bipartite graphs. The following problem could generate future research directions in the study of counting graph homomorphisms.

**Problem 1.**  *Prove or disprove Conjecture 1.*

We also consider another important generalization of the concept of independent sets, dissociation sets. The study of dissociation sets has applications in networking security, wireless sensor networks, scheduling and telecommunications [19,20]. In this paper, we focus on the problem of counting dissociation sets in bipartite graphs. But, the upper bounds given in Theorem 5 and Corollary 2 are not tight. Much more work needs to be done in the future.

**Problem 2.**  *For d≥3, find a tight upper bound on the number of dissociation sets in an n-vertex d-regular graph.*

Another contribution of our work is the simplification of Sason’s [4] entropy approach that can deal with irregular bipartite graphs. But it’s a pity that the entropy approach presented in this paper is not suitable for general graphs. A future work needs to be done that extends the entropy approach to deal with general graphs.

## Data Availability

Not applicable.
